# Supplementary material for: Maternal Resources, Pregnancy Concerns, and Biological Factors Associated to Birth Weight and Psychological Health
Source: J Clin Med. 2021 Feb 10;10(4):695. doi: 10.3390/jcm10040695 (PMC7916643; doi:10.3390/jcm10040695)
Supplement: Supplementary file 1 [file jcm-10-00695-s001.pdf]

Journal of
Clinical Medicine

MDPI

Table S1. Descriptive and correlations between psychological variables.

Variable	Mean	SD	1	2	3	4	5	6	7	8	9	10
1. Negative Affectivity [1]	2.0	0.7										
2. Anxiety [1]	0.9	0.5	0.7*									
3. Optimism [1]	3.5	0.7	−0.2*	−0.4*								
4. Resilience [1]	6.0	0.6	−0.3*	−0.4*	0.4*							
5. Family-Work Conflict [2]	0.9	0.7	0.2*	0.3*	−0.1	−0.04						
6. Pregnancy Concerns [2]	1.7	0.5	0.3*	0.4*	−0.2*	-0.2	0.3*					
7. General Resources [2]	2.1	0.7	0.2*	0.2*	−0.1	−0.3*	0.2*	0.3*				
8. Life Satisfaction [2]	5.5	0.9	−0.1	−0.2	0.2*	0.3*	−0.3	−0.3*	−0.4*			
9. Depression [3]	0.8	0.5	0.4*	0.3*	−0.2	−0.2*	0.2	0.3*	0.2*	−0.3*		
10. Resilience [3]	5.5	1.5	−0.2*	−0.2	0.2*	0.2*	−0.1	−0.1	−0.1	0.2	−0.6*	
11. Optimism [3]	3.4	0.6	−0.2*	−0.3*	0.7*	0.3*	−0.1	−0.1	−0.2	0.2*	−0.4*	0.4*

Standard Deviation (SD). [1] At 9 week of gestation; [2] at 24 week of gestation; [3] at 36 week of gestation. * *p*-value < 0.05 by Pearson's coefficients.
